# Supplementary material for: Ecological forecasts for marine resource management during climate extremes
Source: Nat Commun. 2023 Dec 5;14:7701. doi: 10.1038/s41467-023-43188-0 (PMC10698027; doi:10.1038/s41467-023-43188-0)
Supplement: Supplementary file 1 — Supplementary information [file 41467_2023_43188_MOESM1_ESM.pdf]

SUPPLEMENTARY MATERIAL

Ecological forecasts for marine resource management during climate extremes

Stephanie Brodie[1,2*], Mercedes Pozo Buil[1,2], Heather Welch[1,2], Steven J. Bograd[1,2], Elliott L. Hazen[1,2], Jarrod A. Santora[3,4], Rachel Seary[1,2,3], Isaac D. Schroeder[1,2], Michael G. Jacox[1,2,5]

[1] Institute of Marine Sciences, University of California Santa Cruz, Monterey, CA, USA
[2] Environmental Research Division, Southwest Fisheries Science Center, National Marine Fisheries Service, National Oceanic and Atmospheric Administration, Monterey, CA, USA
[3] Fisheries Ecology Division, Southwest Fisheries Science Center, National Marine Fisheries Service, National Oceanic and Atmospheric Administration, Santa Cruz, CA, USA.
[4] Department of Applied Math, University of California, 1156 High Street, Santa Cruz, CA, USA.
[5] Physical Sciences Laboratory, Earth System Research Laboratories, National Oceanic and Atmospheric Administration, Boulder, CO, USA
[*] Present address: Environment, Commonwealth and Scientific Industrial and Research Organisation, Brisbane, QLD, Australia

Table S1 Summary of global models used for forecasting. All models sourced from the North American Multi-model ensemble (NMME; Kirtman et al., 2014).

**Short name**	**Full Name**	**Ensemble members**	**Start date**	**Lead months**
CanCM4i	Canadian Center for Climate Modeling Analysis	10	1981-01-01	0.5-11.5
GEM-NEMO	Global Environmental Multiscale - Nucleus for European Modelling of the Ocean	10	1981-01-01	0.5-11.5
GFDL-SPEAR	Geophysical Fluid Dynamics Laboratory-Seamless System for Prediction and Earth System Research	15	1991-01-01	0.5-11.5
NASA-GEOSS2S	National Aeronautics and Space Administration - Goddard Earth Observing System Subseasonal to Seasonal	4	1981-02-01	0.5-8.5
COLA-RSMAS-CCSM4	Center for Ocean Land Atmosphere Research - Rosenstiel School of Marine, Atmospheric, and Earth Science - Community Climate System Model version 4.0	10	1982-01-01	0.5-11.5
NCEP-CFSv2	National Centers for Environmental Prediction - Climate Forecast System Version 2	24	1982-01-01	0.5-9.5

Table S2 Summary table describing the months (grey shading) and lead times (values) for each potential TOTAL closure month (black boxes) for the downscaled forecasts. Downscaled forecasts were only initialized in July or January. Note that July before an August closure has an option to either use a forecast from the July initialization (lead 0.5) or the January initialization (lead 6.5), and in this study we only examine lead 6.5 from the January initialization.

	July Initialisation						January Initialisation							
	Jul	Aug	Sep	Oct	Nov	Dec	Jan	Feb	Mar	Apr	May	Jun	Jul	Aug
**Jun closure**	0.5	1.5	2.5	3.5	4.5	5.5	0.5	1.5	2.5	3.5	4.5			
**Jul closure**	0.5	1.5	2.5	3.5	4.5	5.5	0.5	1.5	2.5	3.5	4.5	5.5		
**Aug closure**	0.5	1.5	2.5	3.5	4.5	5.5	0.5	1.5	2.5	3.5	4.5	5.5	6.5 (0.5)	

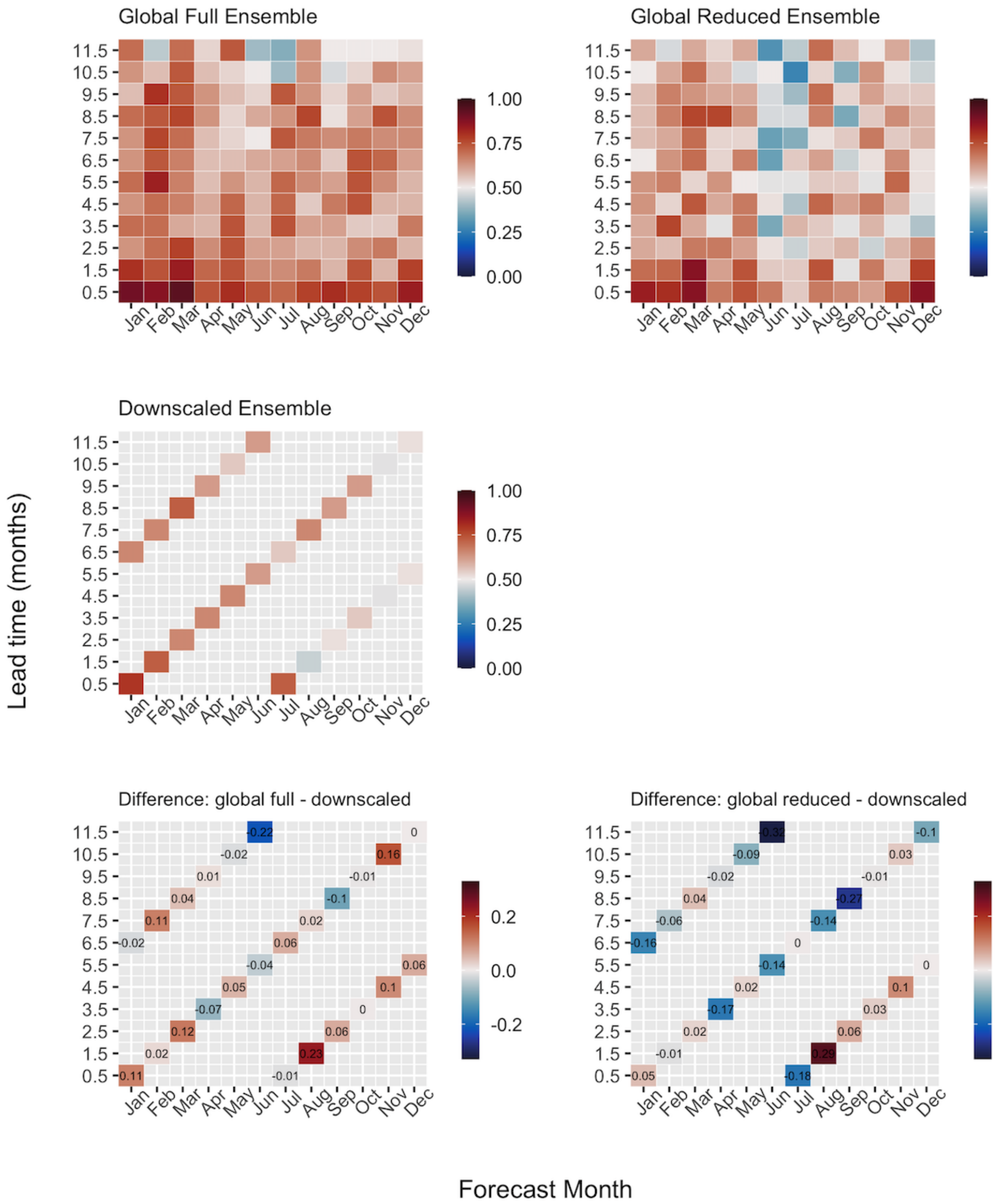

Figure S1 Forecast accuracy of the HCI for the three forecast configurations: Global Full Ensemble (1981-2010), Global Reduced Ensemble (1981-2010), and the Downscaled Ensemble (1981-2010). Bottom row is the difference in forecast accuracy between downscaled and global forecasts, where red colors indicate the global forecast outperformed the downscaled forecast, with blue colors showing the opposite. Source data are provided as a Source Data file.

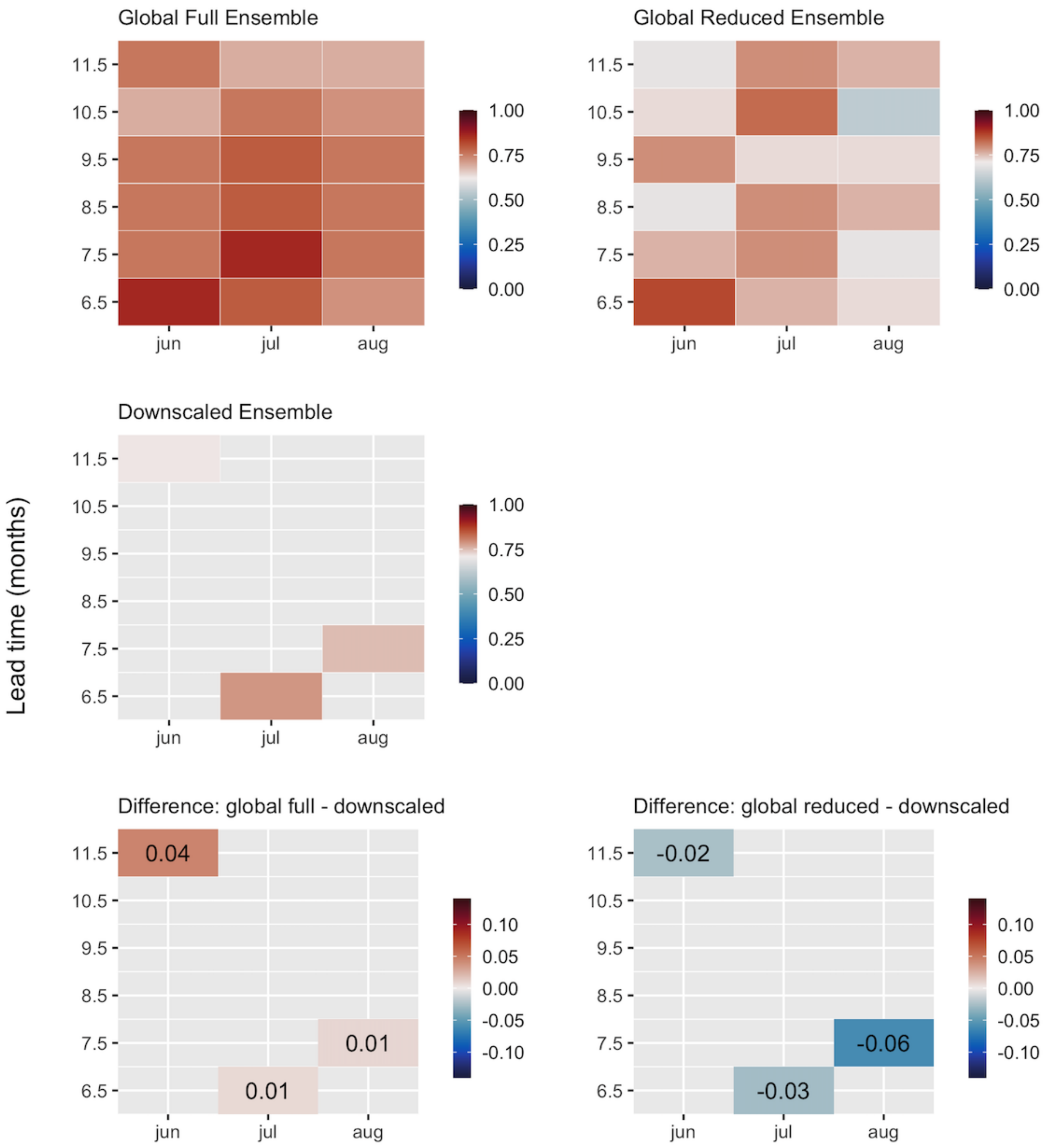

Figure S2 Forecast accuracy of TOTAL for the three forecast configurations: Global Full Ensemble (1981-2010), Global Reduced Ensemble (1981-2010), and the Downscaled Ensemble (1981-2010). Bottom row is the difference in forecast accuracy between downscaled and global forecasts, where red colors indicate the global forecast outperformed the downscaled forecast, with blue colors showing the opposite. Source data are provided as a Source Data file.
