# Supplementary material for: Ecological forecasts for marine resource management during climate extremes
Source: Nat Commun. 2023 Dec 5;14:7701. doi: 10.1038/s41467-023-43188-0 (PMC10698027; doi:10.1038/s41467-023-43188-0)
Supplement: Supplementary file 3 — Reporting Summary [file 41467_2023_43188_MOESM3_ESM.pdf]

nature portfolio

Corresponding author(s): Stephanie Brodie

Last updated by author(s): 11/Oct/23

Reporting Summary

Nature Portfolio wishes to improve the reproducibility of the work that we publish. This form provides structure for consistency and transparency in reporting. For further information on Nature Portfolio policies, see our Editorial Policies and the Editorial Policy Checklist.

Statistics

For all statistical analyses, confirm that the following items are present in the figure legend, table legend, main text, or Methods section.

n/a	Confirmed	
☐	☒	The exact sample size (*n*) for each experimental group/condition, given as a discrete number and unit of measurement
☐	☒	A statement on whether measurements were taken from distinct samples or whether the same sample was measured repeatedly
☐	☒	The statistical test(s) used AND whether they are one- or two-sided<br>*Only common tests should be described solely by name; describe more complex techniques in the Methods section.*
☐	☒	A description of all covariates tested
☐	☒	A description of any assumptions or corrections, such as tests of normality and adjustment for multiple comparisons
☐	☒	A full description of the statistical parameters including central tendency (e.g. means) or other basic estimates (e.g. regression coefficient) AND variation (e.g. standard deviation) or associated estimates of uncertainty (e.g. confidence intervals)
☒	☐	For null hypothesis testing, the test statistic (e.g. *F*, *t*, *r*) with confidence intervals, effect sizes, degrees of freedom and *P* value noted<br>*Give P values as exact values whenever suitable.*
☒	☐	For Bayesian analysis, information on the choice of priors and Markov chain Monte Carlo settings
☒	☐	For hierarchical and complex designs, identification of the appropriate level for tests and full reporting of outcomes
☐	☒	Estimates of effect sizes (e.g. Cohen's *d*, Pearson's *r*), indicating how they were calculated

Our web collection on statistics for biologists contains articles on many of the points above.

Software and code

Policy information about availability of computer code

Data collection	No software was used for data collection
Data analysis	All statistical analyses were completed in R computing software (v4.2.2). Code to make forecasts and figures is available on Github (https://doi.org/10.5281/zenodo.8429074).

For manuscripts utilizing custom algorithms or software that are central to the research but not yet described in published literature, software must be made available to editors and reviewers. We strongly encourage code deposition in a community repository (e.g. GitHub). See the Nature Portfolio guidelines for submitting code & software for further information.

Data

Policy information about availability of data

All manuscripts must include a data availability statement. This statement should provide the following information, where applicable:

- Accession codes, unique identifiers, or web links for publicly available datasets
- A description of any restrictions on data availability
- For clinical datasets or third party data, please ensure that the statement adheres to our policy

Global SST forecasts can be accessed at the North American Multi-Model Ensemble (http://iridl.ldeo.columbia.edu/SOURCES/.Models/.NMME/). Output from the UCSC CCS reanalysis is available from https://oceanmodeling.ucsc.edu. The downscaled SST forecasts and forecasts of HCI and TOTAL generated in this study have been deposited in dryad (DOI: 10.5061/dryad.z08kprrjr). Source data are provided with this paper. The real-time HCI (https://oceanview.pfeg.noaa.gov/whale_indices/) and TOTAL (https://coastwatc h.pfeg.noaa.gov/loggerheads/index.html) management tools are publicly accessible.

Research involving human participants, their data, or biological material

Policy information about studies with human participants or human data. See also policy information about sex, gender (identity/presentation), and sexual orientation and race, ethnicity and racism.

Reporting on sex and gender	Not applicable
Reporting on race, ethnicity, or other socially relevant groupings	Not applicable
Population characteristics	Not applicable
Recruitment	Not applicable
Ethics oversight	Not applicable

Note that full information on the approval of the study protocol must also be provided in the manuscript.

Field-specific reporting

Please select the one below that is the best fit for your research. If you are not sure, read the appropriate sections before making your selection.

☐ Life sciences ☐ Behavioural & social sciences ☒ Ecological, evolutionary & environmental sciences

For a reference copy of the document with all sections, see nature.com/documents/nr-reporting-summary-flat.pdf

Ecological, evolutionary & environmental sciences study design

All studies must disclose on these points even when the disclosure is negative.

Study description	We use two case studies to explore the potential for transitioning existing management tools from nowcast application to forecasting application using downscaled (~10 km resolution) and global (~100 km resolution) model forecasts with 1-12 months lead time.We first conducted a retrospective forecast using global forecasts (73 ensemble members) across the full historically available period (1981-2020) – termed the Global model. We then compared the performance of three forecast configurations: First, we used global forecasts (73 ensemble members) across a reduced historical period (1981-2010) - termed the Global Full Ensemble. Second, we used forecasts regionally downscaled (3 ensemble members) to the CCE for the same reduced historical period (1981-2010) - termed the Downscaled Ensemble. Third, we used a reduced subset of the global forecasts (3 ensemble members) for the same reduced historical period (1981-2010) - termed the Global Reduced Ensemble. All forecasts are compared to SST observations, extracted from a CCE regional reanalysis. This reanalysis is based on the Regional Ocean Modeling System (ROMS) and covers the west coast of the U.S. (30-48˚N, 134-115.5˚W) with 0.1 degree (~10 km) horizontal resolution and 42 terrain-following vertical levels.
Research sample	We use existing ecological indicators designed to mitigate whale entanglements and sea turtle bycatch in the California Current System. The ecological indicators are based on ocean temperatures, and we use global sea surface temperature forecasts from the the North American Multi-Model Ensemble (NMME).
Sampling strategy	We use a retrospective forecast from 1981-2020 to test the accuracy of our forecasts. We use 6 global sea surface temperature forecast models. These six models were chosen as they have multidecadal retrospective forecasts with which we can assess skill and are currently active in the North American Multi-Model Ensemble (NMME).
Data collection	Global sea surface temperature (SST) forecasts are served by the North American Multi-Model Ensemble (NMME); downscaled SST forecasts were produced by paper authors; observed SST were from a data-assimilative regional ocean modeling system (ROMS).
Timing and spatial scale	Global SST forecasts: monthly means, 1981-2020, 1 degree resolution.<br><br>Downscaled SST forecasts: monthly means, 1981-2010. Forecasts not produced for years >2010. Forecasts cover the west coast of

the U.S. (30-48˚N, 134-115.5˚W) with 0.1 degree (~10 km) horizontal resolution.

Observed SST: monthly means, 1981-2020, extracted from a regional reanalysis based on the Regional Ocean Modeling System (ROMS) and covers the west coast of the U.S. (30-48˚N, 134-115.5˚W) with 0.1 degree (~10 km) horizontal resolution and 42 terrain-following vertical levels.

Data exclusions	No data was excluded.
Reproducibility	Data used in analysis is made available on dryad (DOI: 10.5061/dryad.z08kprrjr). Source data for figures is shared. Code used to make forecasts and figures is available on GitHub (https://doi.org/10.5281/zenodo.8429074).
Randomization	Randomization was not necessary in this study as this study did not consist of a controlled experiment. Observed and forecast sea surface temperatures were used in the analysis, with no randomisation required.
Blinding	Blinding was not necessary in this study as this study did not consist of a controlled experiment. Observed and forecast sea surface temperatures were used in the analysis, with no blinding required.

Did the study involve field work? ☐ Yes ☒ No

Reporting for specific materials, systems and methods

We require information from authors about some types of materials, experimental systems and methods used in many studies. Here, indicate whether each material, system or method listed is relevant to your study. If you are not sure if a list item applies to your research, read the appropriate section before selecting a response.

Materials & experimental systems

n/a	Involved in the study
☒	☐ Antibodies
☒	☐ Eukaryotic cell lines
☒	☐ Palaeontology and archaeology
☒	☐ Animals and other organisms
☒	☐ Clinical data
☒	☐ Dual use research of concern
☒	☐ Plants

Methods

n/a	Involved in the study
☒	☐ ChIP-seq
☒	☐ Flow cytometry
☒	☐ MRI-based neuroimaging
